# THe Biom: a platform for visualization and exploration of cancer transcriptomic biomarkers identified by robust feature selection

**DOI:** 10.1093/bioadv/vbag065

**Published:** 2026-02-24

**Authors:** Milan Picard, Elsa Claude, Frédéric Lalanne, Mickaël Leclercq, Raluca Uricaru, Patricia Thébault, Arnaud Droit

**Affiliations:** Département de médecine moléculaire, Faculté de médecine, Université Laval, Québec, QC, Canada; Axe Endo-Nephro, Centre de recherche du CHU de Québec-Université Laval, Québec, QC, Canada; Département de médecine moléculaire, Faculté de médecine, Université Laval, Québec, QC, Canada; Axe Endo-Nephro, Centre de recherche du CHU de Québec-Université Laval, Québec, QC, Canada; Université Bordeaux, CNRS, Bordeaux INP, LaBRI, UMR 5800 Talence, Nouvelle-Aquitaine, France; Université Bordeaux, CNRS, Bordeaux INP, LaBRI, UMR 5800 Talence, Nouvelle-Aquitaine, France; Département de médecine moléculaire, Faculté de médecine, Université Laval, Québec, QC, Canada; Axe Endo-Nephro, Centre de recherche du CHU de Québec-Université Laval, Québec, QC, Canada; Université Bordeaux, CNRS, Bordeaux INP, LaBRI, UMR 5800 Talence, Nouvelle-Aquitaine, France; Université Bordeaux, CNRS, Bordeaux INP, LaBRI, UMR 5800 Talence, Nouvelle-Aquitaine, France; Département de médecine moléculaire, Faculté de médecine, Université Laval, Québec, QC, Canada; Axe Endo-Nephro, Centre de recherche du CHU de Québec-Université Laval, Québec, QC, Canada; Inria, Maasai team-Université Côte d'Azur, Nice, France

## Abstract

The identification of robust transcriptomic biomarkers remains a key challenge in oncology. To tackle this problem, hybrid ensemble feature selection (HEFS) methods have been developed to improve the stability of gene signatures by combining multiple algorithms and data perturbations. However, their results are often difficult to explore, interpret and reuse. To bridge this gap, we developed THe Biom (TCGA HEFS Biomarkers), an interactive application for visualization and comparative analysis of gene signatures across tumor stages and cancer types. The platform enables users to examine cancer-specific biomarkers, track changes across disease progression, and highlight shared features among signatures. THe Biom was built using previous HEFS analyses of six TCGA cancers across stages I to IV, and additional signatures can be added by users.

*Availability and implementation*: THe Biom is freely available online at https://thebiom.compbio.ulaval.ca/, or for local use along with source code and datasets at https://github.com/MilanPicard/the_biom.

## 1 Introduction

The identification of robust transcriptomic biomarkers remains a major challenge in oncology, particularly due to the heterogeneity of tumors and the high dimensionality of gene expression data (Das et al. 2023). Ensemble-based feature selection (EFS) strategies have shown great promise for addressing these challenges. Representing each gene as a variable, EFS strategies train multiple models on the data to identify the most relevant features without relying on a single algorithm which may be sensitive to data noise, bias or sampling variation. Once the outputs are aggregated using various approaches, the resulting selected features are generally more stable and more predictive, often leading to biologically meaningful signatures (Abeel et al. 2009; He and Yu 2010; Zolfaghari et al. 2023). Ensemble-based feature selection has been used successfully to identify prognostic biomarkers on breast cancer (Li et al. 2023), lung adenocarcinoma (Abdelwahab et al. 2022), and renal cell carcinoma (Xin et al. 2024).

Recently, a new approach called hybrid ensemble feature selection (HEFS) has been proposed, which combines data-driven perturbations on top of algorithmic perturbations (Chiew et al. 2019). Data perturbation relies on sampling the dataset into multiple subsets, often using stratified sampling to keep class ratios across runs or other bootstrap sampling techniques with or without replacement. On each subset, various feature selection methods are used to identify the best features. The features that are often found across all perturbations represent the gene biomarkers most likely to succeed in other cohorts and future studies. The HEFS approach, while computationally more intensive, has been shown to repeatedly improve stability and biological relevance of signatures derived from transcriptomic datasets (Cheng et al. 2021; Colombelli et al. 2022).

In our previous work, we introduced a HEFS approach to derive robust gene signatures from RNA-seq data across several cancer types and disease stages (Claude et al. 2024). Rather than simply applying HEFS, we systematically evaluated four distinct HEFS configurations combining filtering methods (differential expression and variance-based) and resampling schemes (stratified repeated holdouts and random stratified sampling). This methodology identified robust and generalizable biomarkers for several cancers including colorectal, renal, pulmonary, and endometrial cancers. While our HEFS framework addressed key methodological gaps in feature selection and biomarker discovery, a critical next step is to make such results accessible to biologists and clinicians, and to facilitate their visualization and their interpretation in specific contexts.

To this end, we developed THe Biom (TCGA HEFS Biomarkers), an interactive web platform providing access to an atlas of HEFS-derived transcriptomic signatures. The platform integrates biomarker gene lists identified with our HEFS framework with multiple visualization approaches and supporting biological context to facilitate interpretation. It enables exploration across cancer types and disease stages, while preserving cohort-specific information through a late-integration strategy based on horizontal data aggregation. THe Biom also supports the integration of user-provided signatures obtained by other methods. The atlas and the utility of THe Biom are illustrated through three case studies demonstrating how they can reveal biologically meaningful patterns across tissues and stages of cancer. Specifically, we explore (i) the identification of transcriptomic biomarkers specific to a particular stage of cancer, (ii) the discovery of common molecular features across stages of the same cancer, (iii) the integration of other signatures and pan-cancer exploration. These use cases highlight the power of the HEFS approach combined with interactive visualization in revealing context-dependent biomarkers, and in supporting hypothesis generation in translational oncology.

## 2 Materials and methods

### 2.1 Datasets

To construct THe Biom atlas, transcriptomic data from The Cancer Genome Atlas (TCGA) were curated for multiple cancer types. Six tumor types were selected to represent a diverse range of tissues and progression profiles: uterine corpus endometrial carcinoma (UCEC), breast invasive carcinoma (BRCA), liver hepatocellular carcinoma (LIHC), lung adenocarcinoma (LUAD), kidney renal clear cell carcinoma (KIRC), and head and neck squamous cell carcinoma (HNSC). For each cohort, we retained both tumor and normal samples, focusing specifically on tumor stages I to IV as defined by the UICC and AJCC classification systems.

Preprocessing of raw RNA-seq data to Transcripts Per Million (TPM) was performed following a standardized pipeline as presented in our previous work (Claude et al. 2024). Quality control filters were applied uniformly across cohorts, including a minimum tumor cell content of 70% for tumor samples, exclusion of formalin-fixed paraffin-embedded (FFPE) tissues, and retention of samples with complete clinical metadata, including tumor staging information. TPM values were log-transformed using log_2_ (TPM + 1) to stabilize variance and enable comparability for visualization and downstream analyses within the THe Biom platform. Samples with insufficient representation in specific categories (e.g., LIHC stage IV, *n *= 4) were excluded from subsequent analyses (Table 1).

**Table 1:** Number of samples per cancer type and tumor stage (I–IV) from the TCGA datasets used in THe Biom project.

Cancer	Normal	I	II	III	IV
UCEC	19	310	48	108	28
BRCA	105	170	574	227	19
LIHC	48	157	75	79	–
LUAD	45	186	81	58	18
KIRC	69	262	57	122	83
HNSC	42	24	62	71	228

### 2.2 Biomarker identification

To identify gene biomarkers distinguishing tumor stages from normal tissues, we used an approach based on Hybrid Ensemble Feature Selection (HEFS) strategy developed by Claude et al. (Claude et al. 2024)designed to enhance the robustness and reproducibility of gene selection in high-dimensional transcriptomic datasets. First, a dimension reduction was performed by applying either a differentially expressed genes analysis (DEG), or an analysis of variance (Var). Then, the HEFS strategy was used, which consists of combining feature selection methods (model perturbations) and various sampling methods (data perturbations) to best identify biomarkers.

These candidate features were evaluated using a set of machine learning models trained to distinguish each tumor stage from normal tissue. Feature importance was computed within each model, and rankings were aggregated across models and resampling iterations using consensus-based methods. Final gene sets were defined by selecting features that consistently ranked among the top across a high proportion (typically ≥70%) of perturbation instances.

For each tumor stage and cancer type, three types of signatures were produced:

- DEG signatures, derived from differential expression
- Var signatures, based on expression variability
- Merge signatures, corresponding to the union of the DEG and Var gene sets.

The HEFS framework supports inter-stage comparisons (e.g., stage II vs. stage III), but preliminary analyses across selected cancer types (LUAD, HNSC, KIRC, and BLCA) did not consistently identify biomarkers during data and algorithmic perturbations. Instead of returning unstable signatures, we discarded these comparisons to prioritize reliable biomarkers that could be generalized to other cohorts. Therefore, only stage-versus-normal comparisons were retained in the final version of the atlas. The resulting signatures form the basis of the gene exploration features available through THe Biom platform.

To facilitate biological interpretation of THe Biom signatures, we integrated pathway annotation data into the platform. Gene-to-pathway mappings were obtained from the Reactome database using its RESTful API. All Ensembl gene identifiers in the signature sets were mapped to associated Reactome pathways. These annotations enrich the atlas with contextual information that supports exploratory analyses and hypothesis generation.

### 2.3 Backend architecture

The backend of THe Biom was designed to support interactive exploration, comparison, and export of transcriptomic biomarker signatures in a reproducible and scalable manner. It is implemented in Python using the Dash framework, which enables tight coupling between data processing and interactive visualization, and relies on Flask for routing and file handling. THe Biom is compatible with several browsers including Chrome, Firefox, and Microsoft Edge.

At a high level, the backend manages three main data types: (i) biomarker signatures associated with metadata such as cancer types and tumor stages, (ii) normalized gene expression values, and (iii) gene–pathway annotations derived from Reactome. These datasets are loaded at application startup from structured files and stored in memory to ensure fast access during interactive exploration.

The backend provides a set of query operations that allow the application to retrieve available cancer types, tumor stages, signatures, genes, and pathways, as well as to compute intersections between signatures. These operations support core functionalities of the platform, including filtering, overlap detection, export of gene lists, and redirection to external enrichment tools such as gProfiler. To ensure visual coherence across the application, color schemes and layout-related parameters are computed centrally and reused across views.

User interactions are handled through Dash callbacks that connect interface events such as gene or signature selection to backend data-processing and dynamically update the visualizations available on the platform. The application allows users to import files and upload custom signatures, provided the file is in the correct CSV format. The signatures are then overlapped with the existing signatures within THe Biom and integrated in the same way as in-house signatures. The interface also allows exporting images and downloading data, enabling users to obtain both visualizations and underlying datasets for further analysis. Overall, the backend architecture emphasizes modularity, consistency of data access, and responsiveness.

### 2.4 User architecture

THe Biom is a web-based interactive platform developed to enable the exploration and comparison of transcriptomic biomarker signatures across multiple cancer types and tumor stages. The interface was developed with a strong emphasis on usability, interpretability, and exploratory analysis, enabling users without computational expertise to navigate complex biomarker datasets.

The platform currently integrates 57 precomputed signatures derived from stage-versus-normal comparisons across six TCGA cancer cohorts (BRCA, LUAD, HNSC, KIRC, LIHC, and UCEC). In addition, users can upload and visualize their own gene signatures using the drop-down menu.

The interface follows a layered visualization strategy inspired by Shneiderman’s “overview first, zoom and filter, then details-on-demand” principle (Shneiderman 1996). It is organized into three complementary views:

Global overview: This view presents all available signatures simultaneously, allowing users to identify similarities and differences across cancer types and stages. It shows the overview graph, where signatures are represented as colored nodes to distinguish cancer types, and weighted edges indicate the number of shared biomarkers between signatures.

Signature-focused view: Upon selection of one or multiple signatures, this view enables detailed inspection of the selected biomarkers. The resulting plot represents signatures as colored hulls, with dots representing individual biomarkers. Biomarker genes are represented as black dots, while green dots represent biomarkers shared by multiple signatures, represented by hulls overlapping. These hulls have arbitrary shapes, but with various color codes and stripe patterns to differentiate cancers and cancer stages. Within this view, users can switch between the mono-signature view, which only shows a single signature, and the multi-signature view, which shows every signature selected. Biological pathways are displayed as gray or red triangles depending on the view selected, allowing functional context to be explored directly. The position of triangles is determined randomly and can be changed by the user by clicking and dragging.

Expression-level detail view: For selected genes, expression distributions across tumor stages are displayed using interactive boxplots. This allows users to assess expression variability and stage-specific patterns, complementing the signature-level information.

Through the drop-down menu, interactive controls allow users to filter signatures by cancer type, tumor stage, genes, or pathways. From the detailed view, selected gene sets can be exported, copied to the clipboard, or directly submitted to gProfiler for functional enrichment analysis through the dynamic legend. Figures generated within the interface can also be downloaded using the menu and were used to produce all visualizations shown in this manuscript.

Together, these interface components provide an intuitive workflow for moving from global comparisons to fine-grained inspection of individual biomarkers, supporting hypothesis generation and biological interpretation of transcriptomic signatures.

### 2.5 Additional signatures

Additional data were gathered to demonstrate the ability of THe Biom to integrate external signatures. We included stage-specific gene sets from three independent studies. (1) colorectal cancer (stages I–IV) from Rahiminejad and al. (Rahiminejad et al. 2022), the second most common cause of cancer mortality worldwide (Ferlay et al. 2022). 110 stage-specific biomarkers were identified through using differentially expressed genes. (2) Breast cancer signatures from Athira et al. were identified for stages I to III using machine learning for a total of 134 biomarkers (Athira and Gopakumar 2022). These signatures were initially selected to compare to our own signature on beast on BRCA cell line, but no overlap was discovered. Finally (3), signatures for stages I to IV for prostate cancer were also selected from Hamzeh et al. for a total of 26 stage specific biomarkers(Hamzeh et al. 2019). Prostate cancer is extremely common in men and is widely studied within the scientific and medical communities (Elmarakeby et al. 2021; Yang et al. 2022; Picard et al. 2024).

## 3 Results

To demonstrate the utility of THe Biom for exploring transcriptomic biomarkers, we selected three case studies that reflect common research needs in signature exploration. These examples were chosen to illustrate the platform’s capacity to (i) identify molecular features specific to a single cancer type and stage, (ii) explore changes in biomarker composition across disease progression, (iii) pan-cancer analysis with integration of external signatures. The selected case studies cover liver hepatocellular carcinoma (LIHC), lung adenocarcinoma (LUAD), and multiple cancer types across stages I to IV. Each case study is centered on a biological question and provides the workflow that users can reproduce or adapt to their own signature analyses.

### Case study 1: Stage II liver cancer biomarkers

The TCGA LIHC cohort focuses on liver cancer, specifically hepatocellular carcinoma (HCC), which accounts for 90% of all liver cancer cases and represents the fourth leading cause of cancer-related deaths worldwide(Samant et al. 2021). Despite the identification of several risk factors, the molecular mechanisms underlying HCC are not fully understood, and additional hypotheses are needed to advance research into its pathogenesis. Here, we illustrate stage II liver cancer biomarkers previously identified by HEFS, representing an early phase of tumor development.

**Figure 1:**
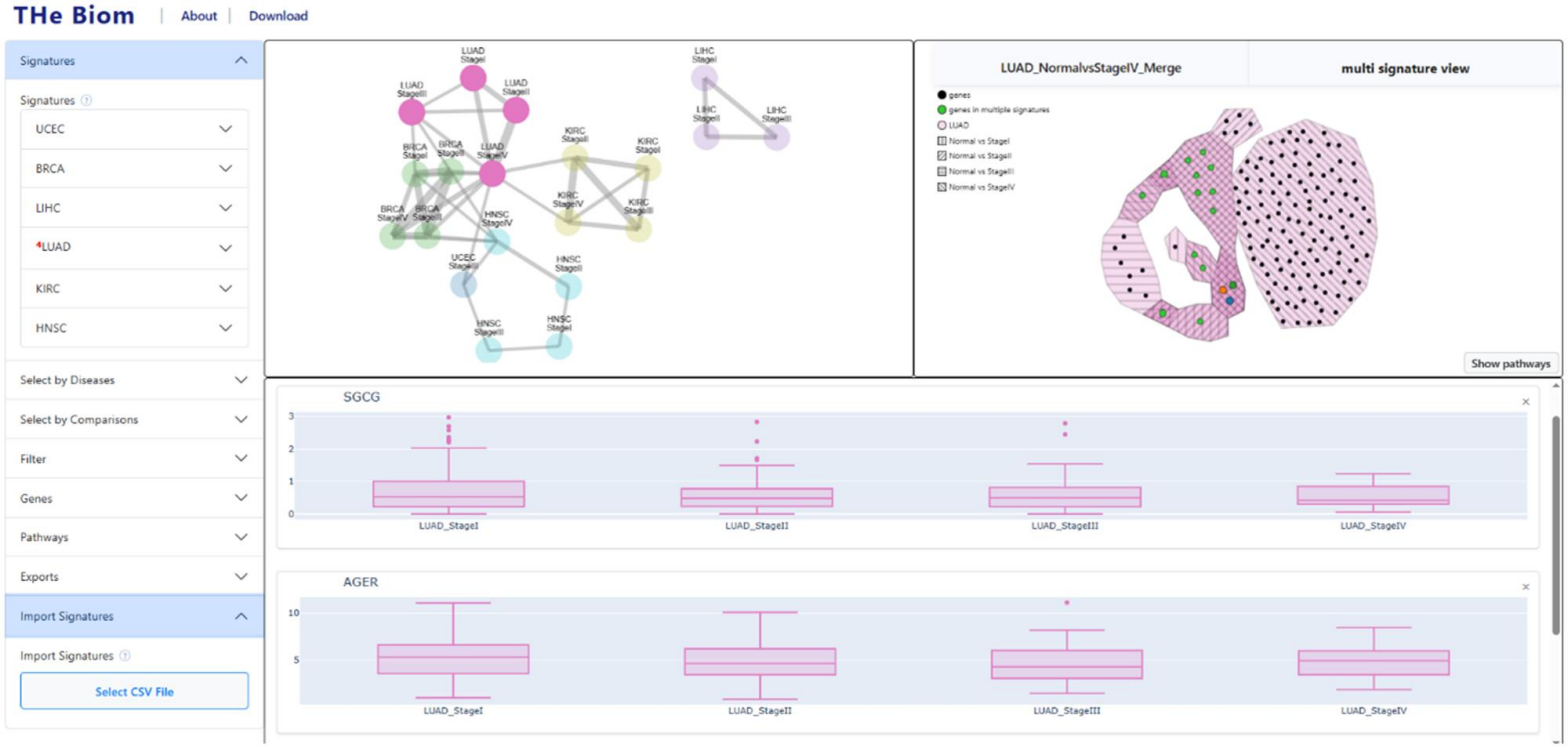
Overview of the main page. It is composed of different tabs, a dropdown menu to filter data (left), an overview graph presenting all available signatures (upper left), a signature-focused view (upper right), and a gene expression view (bottom).

Using THe Biom platform, we can visualize the signature generated from the comparison between normal liver tissue and stage II HCC samples. Using the filter menu (see figure 5, Material and methods), we can easily access these biomarkers in the mono-signature view by selecting the element LIHC and NormalvsStageII in the Signatures tab (Figure 1). This signature consists of eleven protein-coding genes: HOXD10, CLEC4M, COLEC10, ISLR, GDF2, BMPER, BMP10, CYP4F22, ANGPTL6, VIPR1, and RSPO3.

To assess the biological relevance of this gene set, we performed an Over-Representation Analysis (ORA) using gProfiler, accessible via a direct link in THe Biom, clickable by hovering on the shell around the black dots (parameters: Bonferroni correction for multiple testing, minimum annotation gene set size = 5, maximum = 5000, *p*-value < 0.05). The gProfiler analysis could also be accessed by selecting all “genes” on the legend of the multi-signature view. Full results are provided in the Supplementary Materials.

This analysis revealed biologically meaningful annotations:

- Gene Ontology (GO) insights: Several annotations related to the regulation of SMAD and BMP signaling pathways were significantly enriched. These regulatory pathways are known to play key roles in carcinogenesis, particularly in hepatocellular carcinoma, where their dysregulation has been well documented (Hernanda et al. 2015; Zhang et al. 2016).
- Pathway enrichment from Reactome: The pathway Signaling by BMP was also significantly enriched, aligning with the GO findings.

Through THe Biom, users can explore Reactome annotations that may not reach enrichment significance thresholds but could still hold biological relevance. The tool also highlights highly annotated genes within each signature. For example, CYP4F22 is linked to five biological pathways, reflecting its role in hydroxylating fatty acids and eicosanoids. While typically involved in lipid barrier formation in the skin and in the metabolism of inflammatory mediators, the presence of CYP4F22 as a biomarker suggests a potentially central role in liver cancer biology, similar to other fatty acid–metabolizing cytochromes.

### Case study 2: Lung adenocarcinoma (LUAD) progression

Users interested in identifying cancer-specific biomarkers and biological pathways, without restricting the analysis to a particular disease stage, can select multiple stages from the dropdown menu and explore shared similarities between signatures provided in the application. Four signatures were generated by comparing normal lung tissue samples to those from each stage of lung adenocarcinoma progression (LUAD). These signatures contain 8 genes (Stage I), 18 (Stage II), 10 (Stage III), and 136 (Stage IV).

**Figure 2:**
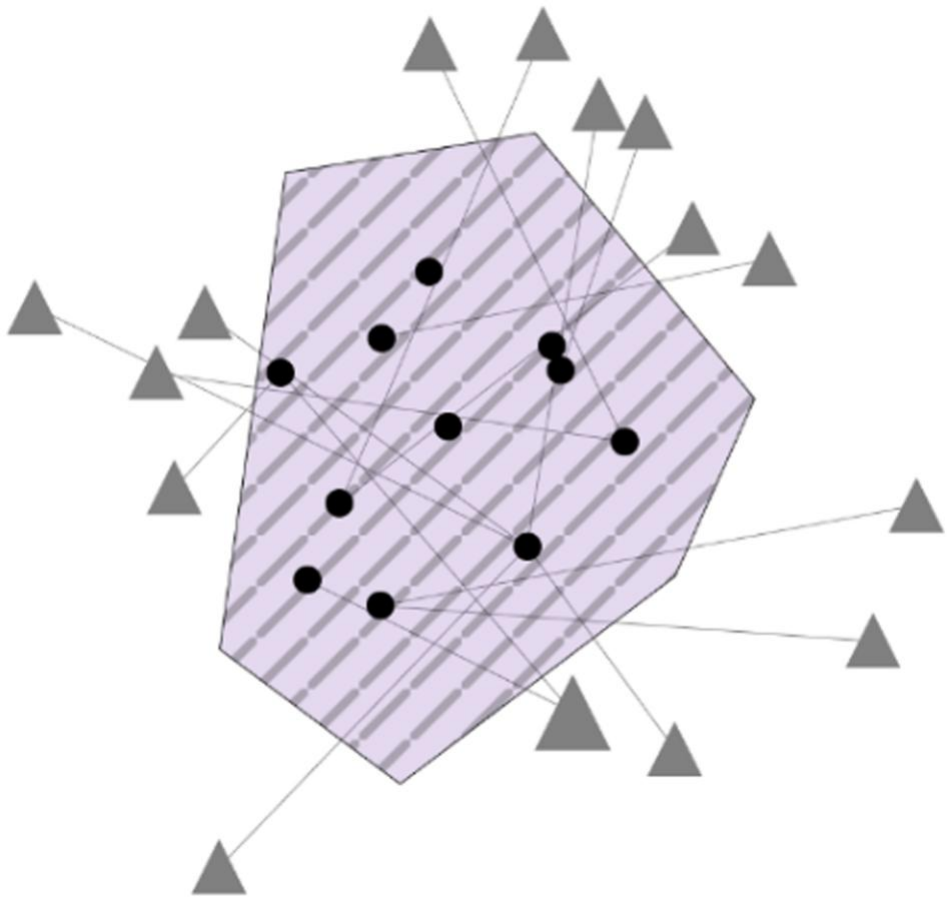
Mono-signature view of the merge signature of LIHC cancer between stage 2 and normal samples. Black dots represent biomarker genes; gray triangles represent associated pathways. The color and stripe patterns of the husk are specific to the cancer and stage selected. The type of signature selected is “merge”, see Materials and Methods.

**Figure 3:**
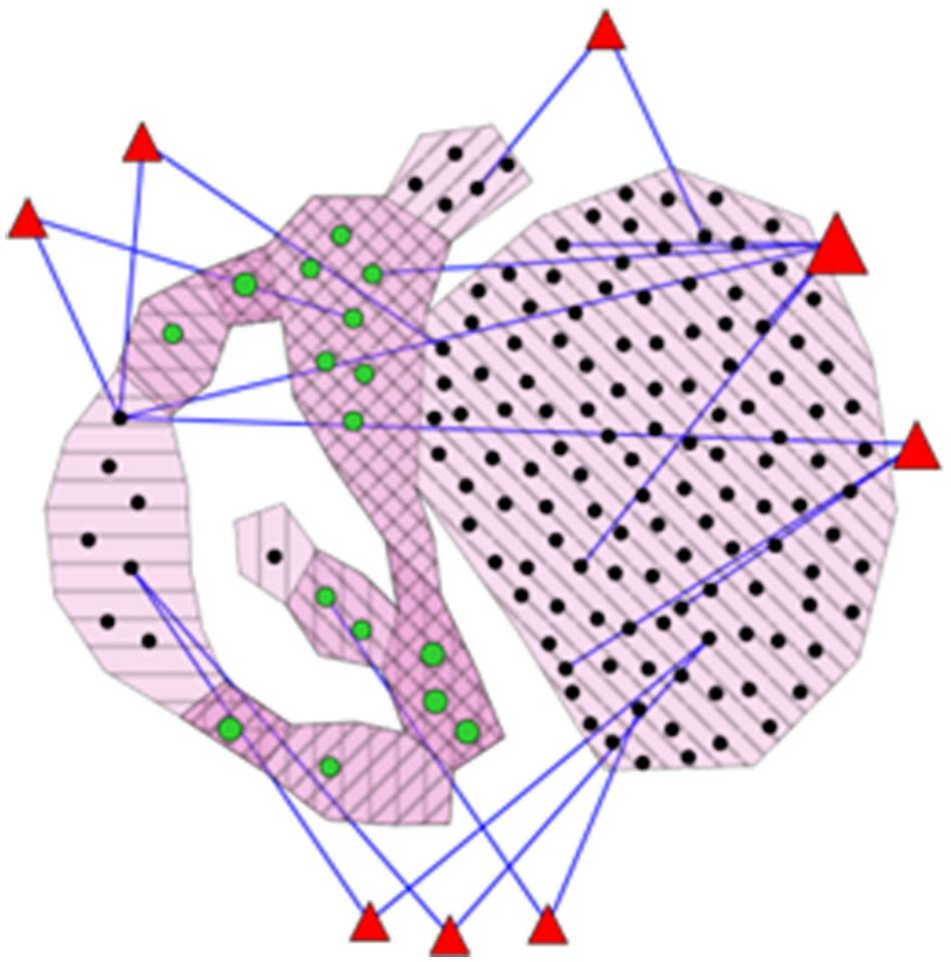
This view shows the signatures available for the four stages of Lung adenocarcinoma (LUAD), as indicated by the stripe patterns. Biomarkers shared between stages are shown in green, with over-lapping stripe patterns. In the multi-signature view, biological pathways are represented by red triangles and are shown only if shared by at least two signatures.

For a detailed comparison across all four stages, users can use the multi-signature view (Figure 2). In this visualization, each type of hull corresponds to different signatures and their overlap, with stages differentiated by a stripe pattern. Green nodes are biomarkers shared by at least two signatures, which can be seen by the number of stripe patterns around them. The figure reveals four regions of double overlap (11 biomarkers), and three regions of triple overlap (5 biomarkers), noticeable by a darker pink color. Compared to other methods for visualizing overlaps such as UpSet plots or Venn diagrams, this visualization method allows users to view each instance (biomarkers) separately and interact with them directly, instead of simply having the size of the overlaps. This interactivity allows the user to directly investigate sets of biomarkers they find interesting through the available pathway information or gProfiler links generated automatically (Figure 3).

Interestingly, one biological pathway stands out: platelet degranulation, which is associated with five genes in LUAD. These include ACTN2 (specific to Stage III), VEGFD, A2M, and GAS6 (specific to Stage IV), and CLEC3B (shared by Stages II and IV). The involvement of platelets in lung adenocarcinoma has been highlighted in recent research, particularly in the context of metastasis dissemination (Hyslop et al. 2020). The fact that three of these genes appear in the most advanced cancer stage supports the hypothesis that platelet activity may contribute to disease progression.

Using the clickable legend of the multi-view signatures, a user can select all biomarkers in common (green nodes) by clicking on “genes in multiple signatures.” The outlines of the corresponding genes will turn orange, and a small tooltip will appear allowing users to do an enrichment analysis via the gProfiler website (Supplementary Materials). Enrichment analysis of LUAD-associated genes revealed key signaling and trafficking complexes. Notably, EGFR signaling, a well-known driver in LUAD, especially among non-smokers, was enriched, consistent with its role in proliferation and survival (Liu et al. 2017). Complexes involving CIN85, SH3GL3, CBL, and MET were also enriched. These regulate receptor endocytosis and degradation, and their dysregulation were also linked to various hallmarks of cancer, lung cancer especially (Havrylov et al. 2010; Tan et al. 2010; Drilon et al. 2017; Lin et al. 2021).

### Case study 3: Pan-cancer analysis

**Figure 4:**
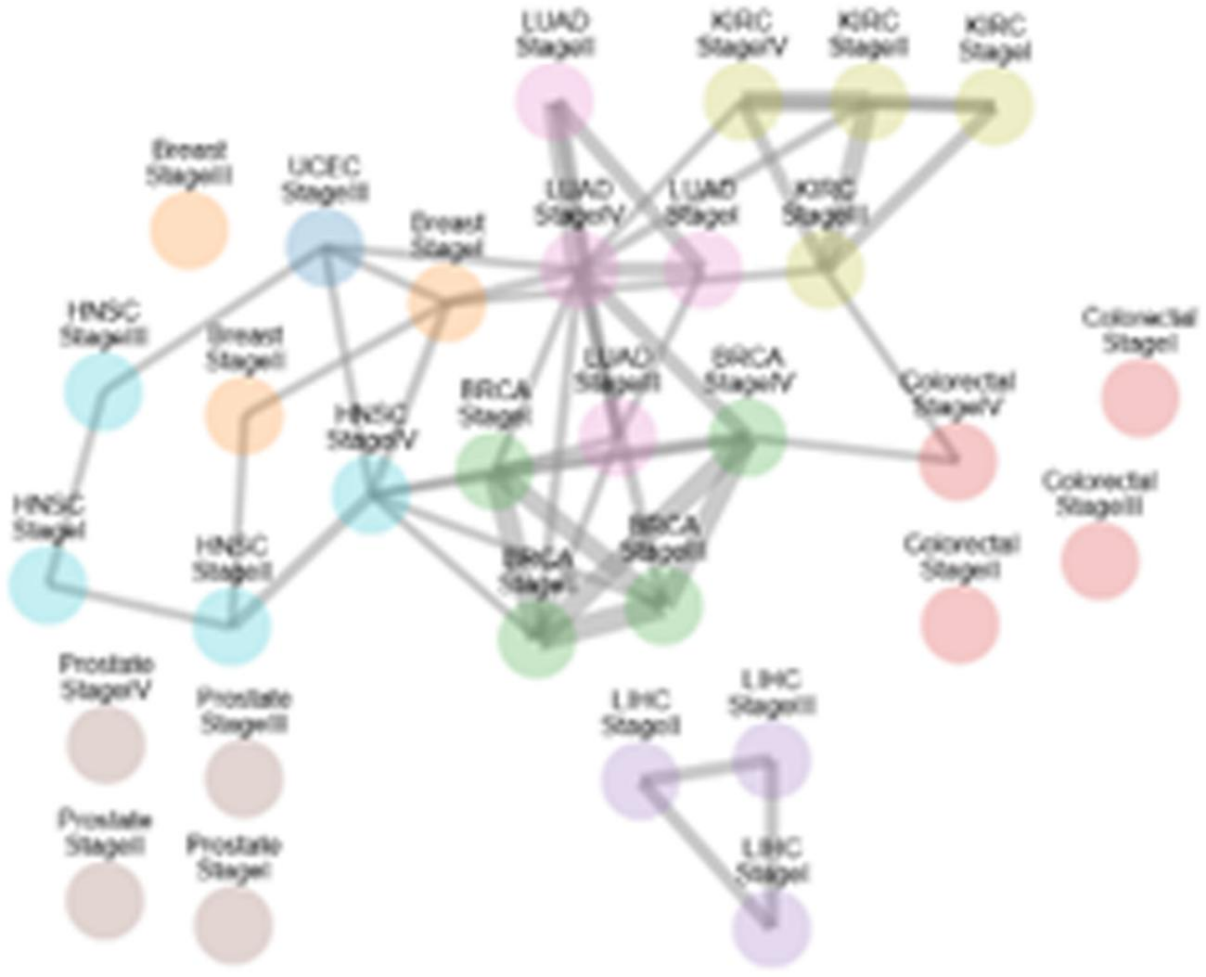
Overview graph of all signatures available in THe Biom, along with the additional signatures of colorectal cancer (stage I to IV), breast cancer (stage I to III) and prostate cancer (stage I to IV). Each node represents a signature. Nodes of the same color represent the same cancer. Weighted edges between nodes represent the number of shared biomarkers between both signatures.

To assess the flexibility of THe Biom in handling external data, we integrated gene signatures derived from independent studies of colorectal cancer (stages I–IV), breast cancer (stages I–III), and prostate cancer (stages I–IV) (Figure 4). Both colorectal and breast cancer signature were identified by comparing each stage to normal tissue, while prostate cancer compared each stage to every other stage. Compared to our pre-calculated HEFS-based signatures, these signatures exhibited much lower overlap, both across stages of the same cancer type and with other cancers. For instance, breast cancer signatures shared only a single biomarker between stages I and II, and colorectal shared a single biomarker between stage I and IV. Prostate cancer signatures had no overlap. This low degree of overlap was unexpected, as stages of the same cancer often exhibit common molecular pathways and shared hallmarks.

**Figure 5:**
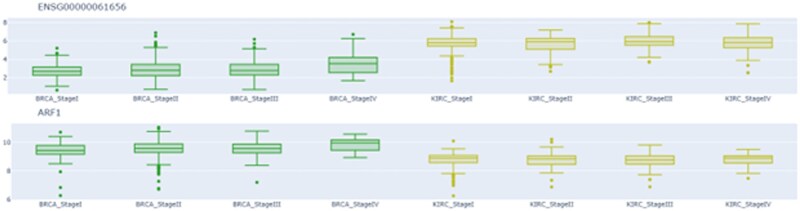
Expression boxplots of ARF1 and SPAG4 (ENSG00000061656) in BRCA and KIRC across cancer stages (I–IV). Expression values are shown as log-transformed TPM.

Despite this, several genes overlapped between these external signatures and ours, including FOXM1, ADH1B, ADIPOQ, TFAP2B, MYL9, GAS6, ARF1, and SPAG4. These shared biomarkers were enriched in pathways such as renal albumin absorption, which is not currently associated with cancer in the literature, and regulation of tumor necrosis factor signaling, a pathway with a well-documented but context-dependent role in tumor progression. While these overlaps did not strongly align with canonical hallmarks of cancer, they illustrate how THe Biom can seamlessly integrate and explore diverse signature sets, offering users the ability to critically examine consistencies, discrepancies, and potential new leads across datasets.

For further insight, we examined the two biomarkers shared between the colorectal cancer signatures, KIRC, and BRCA. These two genes, ARF1 and SPAG4, have been previously reported as a key driver in multiple cancer types (Boulay et al. 2011; Wang et al. 2020) and as a prognostic biomarker in pan-cancer analyses (Zhao et al. 2019). Their expression profiles can be visualized within THe Biom through direct selection of interaction with the detailed network view. Although expression levels do not show marked variation across disease stages, ARF1 displays a modest increase from stage I to stage IV, suggesting a potential association with tumor aggressiveness in breast cancer, consistent with prior observations (Xie et al. 2016). The integration of expression data within THe Biom thus enables direct examination of predicted signatures in relation to disease types and stages, providing an additional layer of biological interpretation.

## 4 Discussion

THe Biom was developed to facilitate the exploration and interpretation of transcriptomic biomarker signatures derived from ensemble feature selection. While methods such as HEFS can produce stable and biologically relevant signatures, their results are often difficult to access or compare without dedicated tools. THe Biom addresses this by providing an interactive platform for visualizing and analyzing signatures across cancer types and disease stages.

The platform uses a late-integration approach that preserves the specificity of each dataset while enabling comparisons between them. This allows users to investigate both cancer-specific and inter-cancer information. The three case studies included illustrate different ways of using the platform, such as identifying stage-specific biomarkers, or examining changes across tumor progression. Crucially, the platform can give biological insights into transcriptomic signatures that the HEFS method might not provide. On top of the biological pathways embedded in the visualization, expression data and easy access to enrichment analysis, THe Biom uniquely provides a coherent and readable way of comparing signatures across various cohorts, bringing context to what would be a plain list of biomarkers. This approach can highlight central and important genes that would have been overlooked without this analysis.

Although the current version includes a limited set of 57 signatures from six cancers, additional signatures could enhance the platform’s coverage and utility. A more diverse signature set would improve the potential to identify shared molecular pathways and support broader comparative analyses. Nevertheless, users can already upload their own gene sets for comparison, allowing the platform to be used beyond its current signature data. This feature may be particularly useful for researchers who wish to reuse the visualization tools of THe Biom on their own signature datasets, making the platform a generalizable tool for researchers and clinicians. Importantly, THe Biom is not confined to HEFS‑derived outputs; it can also integrate signatures produced by any methodological approach, provided they are supplied in the form of a gene list.

Overall, THe Biom provides a lightweight framework for the exploration and comparison of transcriptomic signatures. Its design prioritizes interpretability and usability, with the aim of making computational outputs more accessible to a broader range of researchers working in cancer biology and biomarker discovery.

## 5 Conclusion

THe Biom bridges the gap between complex feature selection outputs and their biological interpretation. By combining interactive visualization, pathway context, and cross-cohort comparison, it transforms gene lists into insights that can be meaningfully explored by researchers. While the current implementation covers a limited number of cancer signatures, its ability to integrate user-provided data ensures flexibility and broad applicability. Beyond serving as a visualization tool, THe Biom encourages dialogue between computational predictions and biological validation, ultimately supporting more informed biomarker discovery and translational cancer research.

## Supplementary Material

vbag065_Supplementary_Data

## Data Availability

The data and code necessary to run THe Biom and reproduce the results are available on https://github.com/MilanPicard/the_biom. The app is also available online at https://thebiom.compbio.ulaval.ca/.
