# Supplementary material for: THe Biom: a platform for visualization and exploration of cancer transcriptomic biomarkers identified by robust feature selection
Source: Bioinform Adv. 2026 Feb 24;6(1):vbag065. doi: 10.1093/bioadv/vbag065 (PMC13032821; doi:10.1093/bioadv/vbag065)

**Supplementary Materials**

THe Biom: a platform for visualization and exploration of cancer transcriptomic biomarkers identified by robust feature selection.

**Case study 1**

Png export from g:Profiler showing the enriched GO terms and pathways for the eleven genes present in the LIHC cancer stage 2 signatures.


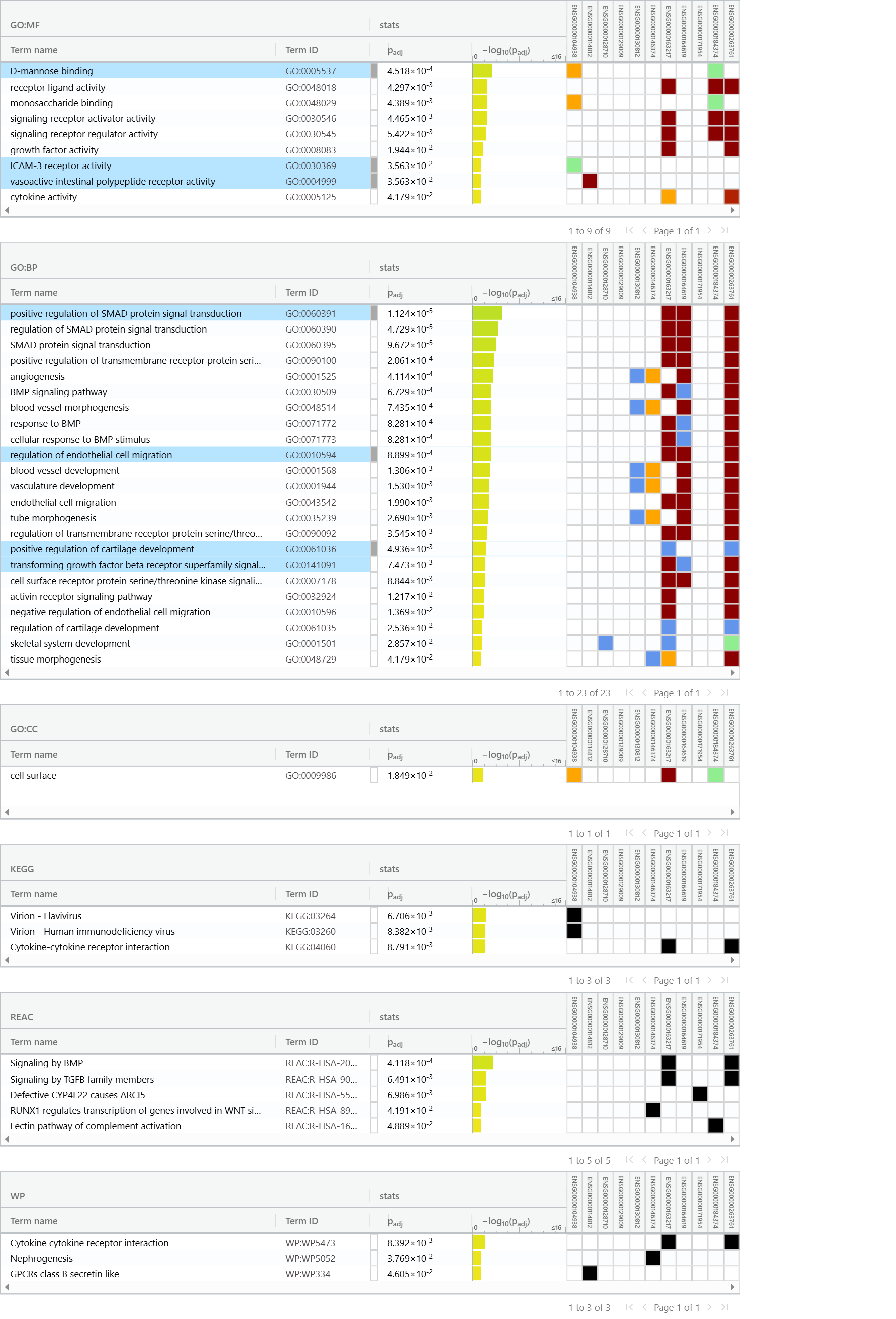


**Case study 2**

Png export from g:Profiler showing the enriched GO terms and pathways for the sixteen genes that are shared between stages of the LUAD cancer signatures.


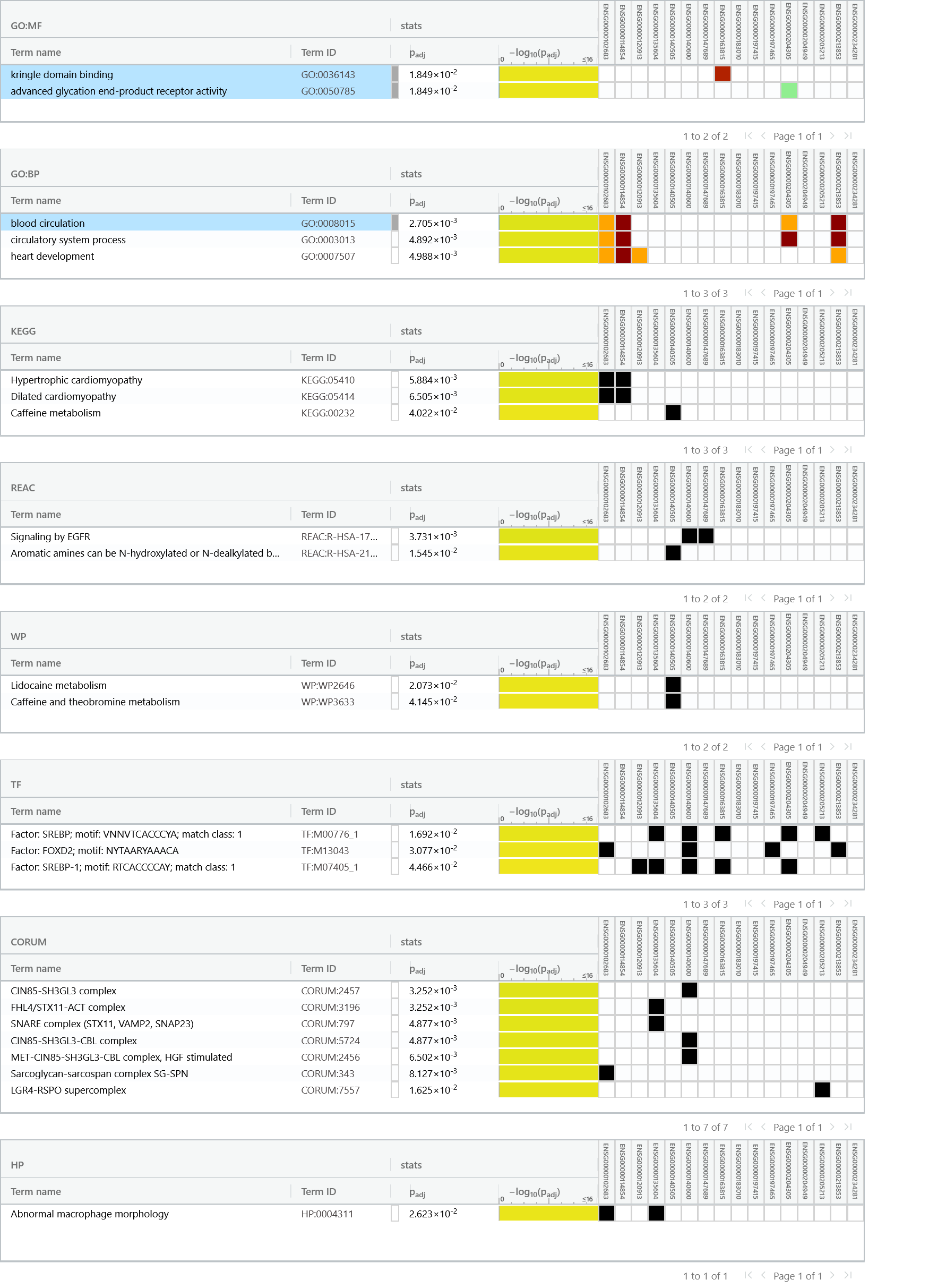

Supplement: vbag065_Supplementary_Data [file vbag065_supplementary_data.docx]
